# Impact of the Domestic Violence Housing First Model on Survivors’ Safety and Housing Stability: Six Month Findings

**DOI:** 10.1007/s10896-022-00381-x

**Published:** 2022-03-22

**Authors:** Cris M. Sullivan, Gabriela López-Zerón, Adam Farero, Oyesola Oluwafunmilayo Ayeni, Cortney Simmons, Danielle Chiaramonte, Mayra Guerrero, Noora Hamdan, Mackenzie Sprecher

**Affiliations:** 1 Michigan State University, East Lansing, MI, USA; 2 University of Michigan, Ann Arbor, MI, USA; 3 Yale University, New Haven, CT, USA; 4 Rutgers University, Camden, Camden, NJ, USA

**Keywords:** Intimate partner violence, Housing, Intervention, Homeless, Economic abuse

## Abstract

Intimate partner violence (IPV) is a leading cause of homelessness, yet little evidence exists about effective strategies to assist IPV survivors as they work to avoid homelessness while freeing themselves from abuse. An ongoing demonstration evaluation is examining if and how one promising model assists IPV survivors in obtaining safe and stable housing over time. The Domestic Violence Housing First (DVHF) model involves providing IPV survivors with mobile advocacy and/or flexible funding, depending on individual needs, in order to attain these goals. We hypothesized that those receiving DVHF would experience greater housing stability and less re-abuse compared to survivors receiving services as usual. The current study evaluated the short-term efficacy of the DVHF model with a sample of 345 homeless or unstably housed survivors who sought services and who completed in-person interviews shortly after contacting the DV agency, as well as six months later. Those who received the DVHF model showed greater improvement in their housing stability compared to those receiving services as usual, as well as decreased economic abuse. Both groups experienced a sharp decline in all forms of abuse. The Domestic Violence Housing First model shows promise in helping unstably housed DV survivors achieve safe and stable housing. Study findings have implications for DV agencies as well as those funding such services. Understanding which interventions work best for which survivors is critical to ensuring that service providers are effectively working toward long-term housing stability and well-being for IPV survivors and their children.

Intimate partner violence (IPV) is a pervasive social problem – approximately one in four women and one in seven men are severely victimized by intimate partners and ex-partners across their lifetimes (Breiding, 2014; Devries et al., 2013). In addition to IPV victimization leading to a wide range of negative financial (Adams et al., 2012, 2020), physical health (Stubbs & Szoeke, 2021), and mental health outcomes (Mason & O’Rinn, 2014; Trevillion et al., 2010), it is also a leading cause of homelessness (Chan et al., 2021; Dillon et al., 2016; Pavao et al., 2007). As a result, DV agencies are increasingly focusing on how they can help unstably housed IPV survivors achieve long-term safe and stable housing (Sullivan & Olsen, 2016; Thomas et al., 2020). One intervention that has grown in popularity but that has not yet been rigorously evaluated is Domestic Violence Housing First, a model purported to increase safe and stable housing by providing IPV survivors with housing-focused mobile advocacy and/or financial assistance, based on their individual needs. The current study is the first to present the impact of this model on IPV survivors’ safety and housing stability across six months.

IPV victimization can result in homelessness and housing instability through direct and indirect pathways. For example, some abusers intentionally damage their victims’ financial and housing stability – by such actions as ruining their credit, stealing from them, preventing them from working, or harassing them at work until they are fired (Adams et al., 2012, 2020; Littwin, 2012; Postmus et al., 2012; Sharp-Jeffs, 2015). Some survivors are forced to frequently move in order to escape abusers who continue to threaten, stalk and harm them (Baker et al., 2003; Long, 2015; Moe & Bell, 2004). The link between IPV and housing instability can also be more indirect. For example, IPV often results in depression or post-traumatic stress disorder (Lacey et al., 2013), which can make it difficult for survivors to engage in day-to-day activities such as going to work or paying bills (Christy et al., 2020; Kimerling et al., 2009; Maddoux et al., 2014). This, in turn, can result in survivors losing their jobs or homes (Adams et al., 2012; Baker et al., 2010).

Domestic violence (DV) victim service agencies in the United States have proliferated in the last 35 years, and there are now over 1800 across the country (National Network to End Domestic Violence [NNEDV], 2021). NNEDV conducts an annual census count of DV services, collecting information about commonly offered services – which continue to include shelter, counseling, legal advocacy, support groups, and children’s programming. For the first time in their 15 years of collecting data, the 2020 census included housing advocacy as a service, reflecting the increased attention paid to this issue by DV agencies. The census found that 46% of DV agencies provided housing advocacy as one of their services (NNEDV, 2021).

While still not widespread, DV victim service agencies are increasingly focusing on helping survivors access safe and stable housing, whether by helping them stay safely in their own homes or assisting them in obtaining new, safe housing (Sullivanet al., 2019a; Sullivan et al., 2019b; Thomas et al., 2020). Advocates working in DV agencies who are helping survivors with housing issues have had to adapt their practices in order to do so (Sullivan et al., 2019a; Sullivan et al., 2019b; Thomas et al., 2020). For example, they have needed to learn how to successfully negotiate with landlords, housing authorities, and other community members who can impact housing availability. In addition to these negotiation skills, they need to know the local, state, and federal housing laws that may help or hinder their clients, and have skills to work creatively with them to obtain resources. This work is difficult, as there is little affordable housing (Shaw, 2020), and many survivors experience housing barriers (which may or may not be related to IPV) that must be addressed. In addition to the IPV-related barriers noted earlier, survivors may also have bad credit (Kofman et al., 2018), criminal records (Engleton et al., 2021; Messing et al., 2015), a problematic rental history (Baker et al., 2010), or other barriers (López-Zerón et al., 2021). Survivors of Color face systemic racism as they seek safe and stable housing (Engleton et al., 2021; Stylianou & Pich, 2019), and some immigrants are unable to work or obtain governmental assistance due to their immigration status (Hernandez-Martinez et al., 2018). These factors coalesce to make the prospect of long-term safe and stable housing less likely.

One promising approach to helping IPV survivors obtain safe and stable housing, which has grown in popularity but lacks rigorous evaluation, is the Domestic Violence Housing First (DVHF) model (Sullivan & Olsen, 2016). DVHF is an adaptation of Housing First (HF), which was initially created to help homeless individuals (primarily single men with either mental health issues or chemical dependency) obtain stable housing (Tsemberis, 2010). HF is predicated on the belief that helping people obtain stable housing before addressing other concerns makes dealing with these other issues more manageable, and the evidence has strongly supported this claim (Greenwood et al., 2020; Padgett et al., 2016).

The HF model has since been adapted to be more applicable to IPV survivors (Sullivan & Olsen, 2016). Adaptations include a greater emphasis on safety concerns and trauma responses, and replacing harm reduction and a recovery orientation with an emphasis on increasing social and emotional well-being (Klein et al., 2021; Sullivan, 2018; Sullivan & Olsen, 2016; Thomas et al., 2020). While HF prioritizes immediately moving people into homes of their own, DVHF considers that some IPV survivors are in extreme danger and would prefer to initially be in a highly secure location such as a DV shelter. A tenet of DVHF is to follow the survivor’s lead in determining when it is safe to be in unsecured housing (Thomas et al., 2020).

A critical component of the DVHF model is that advocates work proactively and creatively with homeless or unstably housed IPV survivors to help them – at their own pace – obtain housing that is not only stable but is also safe. Advocates are mobile, meeting survivors where it is safe and convenient for them, and ideally, advocacy continues as long as survivors need support. In addition to focusing on stable housing, advocates work with survivors on a wide array of areas specific to their individual situations (e.g., safety, employment, immigration).

The other component of DVHF is the provision of flexible funding (Sullivan & Olsen, 2016). Many survivors need financial assistance with issues directly related to housing, such as a security deposit and temporary rental assistance, or help to clear rent arrears (often intentionally caused by the abuser; Adams et al., 2020). Survivors may also need funds that are not typically viewed as impacting housing, but that advocates recognize as critical for housing stability. For instance, survivors may need help obtaining legal documents for housing, or repairing their cars so they can get to work. This flexibility is a fundamental component of the DVHF model and is consistent with the philosophy of DV advocacy to provide survivor-centered services (Cattaneo et al., 2020; Davies & Lyon, 2013). Focusing on increasing survivors’ access to resources is also consistent with Conservation of Resources theory (Hobfoll, 2001), which posits that traumatic life events often result in the loss of economic, social, and interpersonal resources. When this resource loss is followed by resource gain (in this case, housing and safety achieved through housing-focused mobile advocacy and flexible funding), socio-emotional well-being is expected to increase (Sullivan, 2018).

The DVHF model is also grounded in empirical and practice evidence suggesting that mobile advocacy and flexible funding have multiple positive impacts on survivors. Specifically, mobile advocacy has been found to increase survivors’ quality of life, social support, and ability to access community resources, while decreasing their risk of re-abuse and depression (Bybee & Sullivan, 2002; Sullivan & Bybee, 1999). Providing IPV survivors with financial assistance tailored to their individual needs has also been found to lead to housing stability and increased safety (Sullivan et al., 2019a; Sullivan et al., 2019b). Further evidence supporting the importance of mobile advocacy and financial support for DV survivors was demonstrated through a DVHF pilot project, which involved interviewing survivors who had received mobile advocacy and flexible funding from one of nine agencies across one state in the Pacific Northwest (Mbilinyi, 2015). Of those families who could be located and interviewed, the majority reported being effective at accessing and retaining housing at six, twelve, and eighteen months after program entry.

The current study builds on prior evidence by examining the extent to which housing-focused mobile advocacy and flexible funding contribute to increased safety and housing stability for IPV survivors. We hypothesized that survivors receiving mobile advocacy and flexible financial assistance (DVHF) would show greater improvement at the six-month follow-up on these dimensions compared to survivors receiving services as usual (SAU).

## Method

### Study Design

The current study includes baseline and six-month data from an ongoing longitudinal study examining how effective DVHF services are in helping unstably housed IPV survivors obtain safe and stable housing over the course of two years. We intentionally used a naturalistic quasi-experimental study design, with data collection starting when survivors sought services from a DV program. While randomized control trials are ideal for examining intervention outcomes under tightly controlled conditions, they are not designed to capture real-world complexities intrinsic to community services provided to vulnerable populations (Crane et al., 2019; Goodman et al., 2018; Sanson-Fisher et al., 2007). Given the reality of nonprofit DV agencies, there are many times that shelters are full, advocates are overcommitted, and/or flexible funding is limited or unavailable. Survivors are therefore sometimes able to access all the services they need, while other times they may receive only some of the assistance they need or services that do not quite match their needs. Inviting all eligible survivors into the study ensured natural variability in service delivery that allowed for a systematic examination of the DVHF model (for more information on the study design see Chiaramonte et al., 2021). Those who received the DVHF model (housing-focused mobile advocacy and/or flexible funding) were compared to those who received SAU, which may have included counseling, support groups, shelter, information and referrals, safety planning, or other forms of advocacy not related to housing stability (e.g., health-related).

## Participants and Procedures

Participants were recruited from five DV organizations (two urban, three rural) in a state in the Pacific Northwest. Each agency confirmed that they subscribe to the DVHF model but that there are times they do not have the funding nor staffing to provide housing-focused, mobile advocacy. During the time of study recruitment, DV agency staff agreed to inform all new clients who were homeless or unstably housed about the study. They referred 597 clients who were interested in hearing more about the study. We successfully reached 514 of these clients (86%) and told them more about the study. Fifteen percent were ineligible for the study because they either had not experienced recent IPV or were not either homeless or unstably housed. Seven percent declined to participate after hearing more (eight participants specifically noting safety concerns). The final sample consisted of 406 participants (93% of the 438 eligible clients). While agency staff kept no written documentation to support this, they verbally confirmed that few clients were ineligible to participate in the study and that those who enrolled in the study were similar demographically to all their clientele.

Interviews were conducted in English (88%) or Spanish (12%), depending on participant preference. Participants were paid $50 per interview, and all interviews were conducted either in-person or over the phone (also based on participant preference) by highly trained and supervised interviewers. Interviews averaged one hour and fourteen minutes at baseline, and one hour and sixteen minutes at six-months. Institutional Review Board (IRB) research approval was obtained for this study through the first author’s university.

## Measures

In addition to basic demographic data, interviews captured the following information used to examine change over six months on safety and housing stability:

### Services received

The six-month interview included a number of questions about services received, including whether participants had received counseling, support groups, shelter, transitional housing, advocacy, and referrals. They were also asked if a staff member helped them “work on housing and getting other things” they needed from the community.

### Flexible funding received

Each agency documented the provision of financial assistance provided to each study participant on a password-protected, secure online spreadsheet that was shared with the evaluation team. Entries included dates, amounts, and descriptions of how funds were used.

### Safety

Physical abuse, emotional abuse, sexual abuse, and stalking were assessed using a modification of the 28-item Composite Abuse Scale (CAS; Loxton et al., 2013). Two items in the CAS (*hang around outside your house* and *harass you at work)* were replaced with a new item *(repeatedly follow you, phone you, and/or show up at your house/work/other place*) to capture multiple indicators of stalking behaviors that were relevant even if the participant was living with the abuser. Four new items were added to the CAS to address abusive behaviors not adequately measured in the original scale: 1) *stalk you*, 2) *strangle you*, 3) *demand sex whether you wanted to or not*, and 4) *force sexual activity*. Questions were asked within the format: “How often, if at all, did [abuser’s name]…” The original response options for the CAS, which ranged from *daily* to *never*, were modified to accommodate interviews occurring every six months. The response options for the current study ranged from 0—5: 0 = *never*, 1 = *once*, 2 = *several times or between 2–3* × *in the last 6 months*, 3 = *once a month*, 4 = *once a week*, and 5 = *daily*.

The final measure included 31 items across four subscales. Eleven items measured physical abuse (Cronbach’s alpha = 0.91). Thirteen items measured emotional abuse (Cronbach’s alpha = 0.91). Three items measured sexual abuse (Cronbach’s alpha = 0.92). Four items measured stalking/harassment (Cronbach’s alpha = 0.84). Cronbach’s alpha for the full measure was 0.95.

The 14-item Revised Scale of Economic Abuse (SEA2; Adams et al., 2020) measured abusive tactics specifically targeted toward jeopardizing intimate partners' and ex-partners’ economic stability. Sample items included asking how often in the prior six months the abuser would ‘*force or pressure you to give them your savings or other assets*,” and “*keep you from having a job or going to work*.” Response options ranged from 0 = *never* to 4 = *quite often*. Cronbach’s alpha for the measure was 0.91.

### Housing instability

A seven-item Housing Instability Scale (HIS) was created specifically for this study. It included six items from the 10-item The Housing Instability Index (Rollins et al., 2012) as well as an additional item: “In the last 6 months, have you been homeless or had to live with family or friends to avoid being homeless?” Four items from Rollins and colleagues’ (2012) Housing Instability Index were not included because they related to landlords or renting, and many of this study’s participants did not have landlords. Of the seven items in the final scale, five included original dichotomous *yes/no* responses while two items were recoded to be dichotomous. For each item, 0 = more stable and 1 =less stable. Scores range from 0–7, with higher scores indicating greater instability.

To assess the psychometric properties of the HIS in both English and Spanish, we examined measurement invariance, concurrent validity, and predictive validity. The scale demonstrates strong concurrent and predictive validity, and shows evidence of scalar equivalence over time and across both the English and Spanish versions (see Farero et al., 2021). Coefficient alphas for the HIS were examined at each wave of data collection and Cronbach’s alpha for the study was 0.79 (*M* = 3.00, *SD* = 2.24).

## Retention

Retention at six-month follow-up was 92%. The demographics of the participants retained in the study were comparable to those who were not retained regarding age, race, ethnicity, relationship status, number of children, history of abuse, history of homelessness, and mental health symptomatology.

## Analyses

### Determining who received DVHF

We used participant interviews and agency data to determine who had received the DVHF model. First, we removed the 30 participants (8%) who had received no services from the agency at all. Of the remaining 345 participants, slightly more than one-third (36%, n = 124) were categorized as SAU because they had: 1) received services but had not worked with an advocate on housing-related issues, despite reporting needing such help; and 2) had no record of receiving flexible funding. These were typically people who had received counseling, help with restraining orders, information or referrals, shelter services, or were in support groups. Unlike housing-focused mobile advocacy and flexible funding, which are not widely offered, these other services are typical of what IPV survivors can expect from a DV agency.

Survivors who received housing-focused advocacy and/or flexible funding were considered to have received at least some form of DVHF. Sixty-four percent of the service-receiving sample (n = 221/345) received some aspect of the DVHF model (18% received flexible funding but no housing-focused advocacy, 29% received housing-focused advocacy but no flexible funding, and 53% received both).

### Hypothesis testing

Hypotheses were tested using inverse-probability-weighted regression-adjustment (IPWRA) models (Austin & Stuart, 2015), comparing those who received the DVHF model with those receiving SAU. IPWRA addresses potential selection bias in non-randomized intervention studies by simultaneously estimating two models: a ‘treatment’ model that includes factors that increase the probability of receiving the intervention, and an ‘outcome’ model that includes factors associated with the outcomes (e.g., the intervention and other relevant covariates). Because differences between the two groups at baseline could affect outcome trajectories if not controlled for, we first examined whether there were any baseline differences between those who received DVHF and those who received SAU. We used logistic regression models to test whether 72 variables and scales (demographics as well as outcome variables and potential mediator or moderator variables) predicted membership in the DVHF and SAU groups (predictors were examine separately) and found 15 significant associations (all with small differences; see Table 1). Thirteen of these predictors were included in the treatment portion of the IPWRA model. Two factors were omitted: “Seeking help with housing” perfectly predicted cases, which would have resulted in their exclusion from the model; and “Stalking” is a subscale of Overall Abuse (which was included in the model) and the two baseline scores were highly correlated (r = 0.811).

The small but significant differences on the 13 predictors suggest that, generally, those who received DVHF had fewer barriers and greater assets at baseline compared to those in SAU. Survivors who received DVHF were less likely to have lived with their abuser at baseline, were less likely to have been in foster care, less likely to report barriers to housing, less likely to have stayed with friends and family to avoid homelessness, were better able to make ends meet, had experienced less abuse, were less likely to misuse drugs and alcohol, had higher quality of life, and had greater housing stability when compared to those who received SAU. Those in the DVHF group were also more likely to identify as a racial minority, to be parenting children, and to have sought help from one of the urban agencies. Again, all of these differences were small.

The outcome portion of the IPWRA model included the baseline outcome and controlled for twelve covariates that could potentially impact one’s safety and housing stability (e.g., age, race, employment, education). IPWRA models were conducted for each outcome. To account for the natural clustering of our data (survivors nested within advocate nested within agency) cluster-robust standard errors (CR-SEs) were used. Specifically, agency was treated as a fixed effect across all models, and clustering by advocate was accounted for by obtaining standard errors that reflect the nature of these clusters (McNeish & Kelley, 2019; McNeish et al., 2017). All analyses were conducted in Stata 17.

## Results

Demographic information collected at baseline revealed that study participants were predominantly female (97%) and heterosexual (86%). Ages ranged from 19 to 62, with an average of 35 years old. Thirty five percent were non-Hispanic White, and 65% reported a minority racial/ethnic identity: Hispanic/Latinx (35%), Black (19%), US Indigenous (12%), Asian (4%), and/or Middle Eastern (1%). Of the minority survivors, 15% selected more than one race/ethnicity category, indicating multiracial or multi-ethnoracial identities. Most participants (74%) had children they were responsible for raising at the time of the study. See Table 2 for more details about participant socio-demographics.

Participants were asked about their current housing status at each time point. Those who reported living on the street, in their car, in a building not suitable for habitation, in a hotel, or in a shelter were categorized as homeless (42% at baseline). An additional 22% of participants at baseline were staying with family or friends and contributing no rent. The remaining participants were either living in a transitional housing or substance abuse treatment program rent-free (3%), staying with family or friends and contributing to rent (9%), or they rented/owned their own home but were either unsafe there or at risk of losing it (24%).

At the six-month time point, housing stability improved across the entire sample. Only 13 percent of the sample was homeless or living in a shelter (29-point decrease). Those staying with family and friends rent-free decreased slightly (from 22 to 19%), while those living with family and friends and paying partial rent increased (from 9 to 15%). Those in transitional housing or substance abuse treatment programs increased (from 3 to 8%). The largest change was in the number of people renting or owning their own home (increase from 24 to 46%).

## DVHF Impact on Housing Stability

The IPWRA models compared the 221 people who received DVHF with the 124 people who received SAU. These analyses revealed that survivors who received the DVHF model reported significant improvements in housing stability compared to those who had received SAU (*b* = −1.244, *p* < 0.05), with a medium effect size (*d* = 0.565). Table 3 presents the IPWRA models and Table 4 presents group and total means at both time points for all outcome variables.

## DVHF Impact on Safety

Survivors had experienced a range of IPV in the six months prior to seeking services: emotional (95%), physical (91%), economic (91%), stalking/harassment (89%), and sexual (52%). Survivors who received the DVHF model reported significantly less economic abuse than did those receiving SAU (*b* = −0.163, *p* < 0.05), with a small effect size (*d*=0.194). No significant group differences were found for any other form of abuse, and survivors across both groups noted a significant decline in violence between baseline (*M* = 1.69, *SD* = 1.13) and six months (*M* = 0.54, *SD* = 0.73), *t*(339) = 19.00, *p* <0.001) with a large effect size (*d* = 1.03).

## Examining Service Differences by Race and Ethnicity

Although a large, eight-state DV shelter study found no race or ethnicity differences in clients’ views of services received or treatment by staff (Sullivan & Virden, 2017), a qualitative study did uncover the existence of some microaggressions within DV shelters against survivors of Color (Nnawulezi & Sullivan, 2014). We therefore examined whether there were any race or ethnicity differences in the current study with regard to services received. After controlling for agency and advocate, logistic regression results indicated that minority status was not a significant predictor of services received.

## Discussion

This study is the first to rigorously examine the impact of the DVHF model on survivors’ housing stability and safety. As hypothesized, those who received the DVHF model experienced greater housing stability at six months compared to those receiving SAU. Given that a primary goal of DVHF is to assist survivors in stabilizing their housing situations, this is a very promising finding. While the “services as usual” that DV agencies provide may positively impact survivors’ safety and well-being (Sullivan, 2018), the provisions of mobile advocacy and flexible funding appear to be especially salient in achieving stable housing. This finding supports an earlier study that noted improvements in housing stability among IPV survivors who received financial assistance (Sullivan et al., 2019b) as well as a pilot evaluation of the DVHF model (Mbilinyi, 2015).

Survivors receiving the DVHF model also experienced less economic abuse from their abusive partners and ex-partners compared to those receiving SAU. There were no group differences on other forms of abuse, but survivors across both groups noted a significant and steep decline in violence between baseline and six months. This overall decrease in abuse may reflect positively on DV services as a whole, although we cannot definitively conclude this in the absence of study participants who did not seek help. Findings do, however, support prior evidence that DV agency staff make a difference in the lives of survivors (Davies & Lyon, 2013; Goodman & Epstein, 2008; Sabri et al., 2021; Sullivan & Virden, 2017). The steeper decrease in economic abuse for those receiving the DVHF model, however, is notable. Although additional research is needed to confirm the reasons underlying these effects, one plausible explanation is that advocates who are focused on stabilizing housing as part of their work are likely attending to the multiple barriers survivors face in obtaining this objective (Sullivan et al., 2019b). While physical, emotional, and sexual abuse tends to occur ‘behind closed doors,’ economic abuse can be more visible – perhaps making it easier for advocates to help survivors manage and prevent. It is possible that advocates explicitly focused on how the survivors they were working with were experiencing economic abuse, and then individualized their responses accordingly (Adams et al., 2020; Rivas et al., 2019). For example, if a survivor’s abusive ex-partner was calling her place of employment to harass her, the advocate and survivor may have put safety measures in place to prevent this from happening. If a survivor’s abuser stole her paycheck, the advocate might have helped with financial assistance from the DV agency and by implementing new procedures to prevent this from recurring.

It is also possible that the provision of flexible funding contributed at least in part to the decrease in economic abuse. As survivors gained more economic resources under their own control they may have been less vulnerable to the abusive (ex) partner harming them financially. There could also be a bi-directional relationship, whereby as the survivor’s housing situation stabilizes, they are at less risk of needing contact with the person who had been economically abusing them. Less contact provides less opportunity for some forms of economic abuse, such as stealing a person’s credit cards or withholding money from them. Analyzing data from the later time points within the current longitudinal study as they become available may shed additional light on this finding.

After controlling for agency and advocate, our study did not find any race or ethnicity differences regarding services received. Given the inconsistent findings regarding race and ethnicity differences among DV survivors’ access to and receipt of services (Nnawulezi & Sullivan, 2014; Sullivan & Virden, 2017; Waller et al., 2021), future research should continue to delve into this important issue.

## Limitations

Findings should be considered in light of study limitations. All participants in this study were unstably housed or homeless and had sought help from a DV program. Further, while the study included a majority of racial-ethnic minorities, there were few Indigenous participants or those of Asian or Arab descent. Most participants were also cisgender females. As such, the findings are not generalizable to all IPV survivors. Further, the use of self-report data may introduce bias resulting from selective recall or inaccurate self-assessment. There were also practical and ethical reasons why we could not randomize study participants into either receiving DVHF or SAU (for more detail, see Chiaramonte et al., 2021). While we took numerous steps to ensure the accuracy of service classification, and controlled for pre-existing group differences, it is still possible that unidentified relationships may be at play.

The current study only tested whether the DVHF model as a whole led to better housing and safety outcomes than did SAU. However, there was variability in what survivors received within the DVHF model and what they received as SAU, and that complexity has not yet been examined. This initial study also did not examine the two components of the model separately – specifically, whether housing advocacy without flexible funding is more or less effective than flexible funding without housing advocacy, or whether receiving both housing advocacy and funding is more effective than receiving only one. Answering these questions will be complicated, as it is difficult to assess the extent to which services fully matched what survivors wanted and needed across time. Studies with more frequent assessments than every six months will be better suited to evaluating changes in survivors’ needs more dynamically.

Similarly, the current study does not address “dosage” – whether more funds and/or more time with an advocate lead to better outcomes. A tenet of both HF and DVHF is that “more is not always better,” and that service response should only provide what is needed but not more (López-Zerón & Sullivan, 2019; Sullivan & Olsen, 2016). Some people only request a “light touch,” for example, perhaps needing only one-time assistance to respond to a crisis (Culhane et al., 2011; López-Zerón & Sullivan, 2019; Sullivan et al., 2017), while others require far more extensive help to stabilize their lives. Sometimes referred to as “progressive engagement,” the idea is to match the amount of funds and time to individuals’ needs, not only to effectively assist individuals but to judiciously allocate resources, so they are available to the greatest number of people (Culhane et al., 2011; National Alliance to End Homelessness, 2021).

Although our data could identify which survivors received what services, we cannot confidently speak to *why* survivors received what they did. A principle of DV services is to be survivor-driven, offering a wide range of assistance and following the survivor’s lead in what they receive and when they receive it (Cattaneo et al., 2020; Davies & Lyon, 2013; Sullivan & Goodman, 2019). This does not always happen in practice, often due to resource constraints and staff conceptions about what people should receive. Implicit bias is pervasive, for example, and can impact what staff offer or how they work with different people (Holroyd et al., 2017; Wong et al., 2021). In our examination of baseline differences between those who received DVHF and those who received SAU, 15 of the 72 comparisons were significantly different (albeit with small differences). One would expect, simply by chance, that some comparisons would be significantly different, and it is noteworthy that 57 of the comparisons were not significant. However, the significant differences showed that survivors who received DVHF had fewer barriers and greater assets at baseline compared to those in SAU. While these differences were controlled for in outcome analyses so did not impact findings, we cannot determine whether any of these baseline differences impacted either survivor interest in receiving the DVHF model or staff decision-making about what they offered. This speaks to the need for far more research in understanding the circumstances under which services are delivered, and to whom.

Finally, much more needs to be understood about the survivors who received SAU. Some survivors reported that they had wanted housing advocacy but had not received it, but others reported not wanting such help. We do not know if this is because they wanted to stay in their homes and so did not interpret a question about housing advocacy as applying to them, or if they did not feel “ready” to think about housing at the time they were asked the question, or if something else was driving this response. The DVHF model is not necessarily the correct approach to use with every unstably housed DV survivor, and far more needs to be understood about all of the alternatives desired by survivors themselves, as well as how other services lead to positive outcomes.

## Implications

The study findings offer valuable practice implications. Given the scarcity of affordable housing nationwide and the varied and unique needs of survivors, it is critical for programs to offer services tailored to survivors’ specific housing needs. However, helping survivors obtain and maintain stable housing can be time-consuming and complex (Sullivan et al., 2019b). Policymakers and agency leaders must therefore support direct service staff in their efforts to help survivors remove potential housing barriers, find safe housing, and maintain this housing. To do this work, advocates need to build relationships with landlords and housing authorities, gain knowledge about local resources, and have the time and flexibility to provide mobile, survivor-driven advocacy. This suggests that funding of services should not be driven by how *many* people are served but by how *well* they are served, even if fewer people receive services because clients are receiving adequate amounts of time and resources. Additionally, agencies need funds that can be flexibly used to help survivors obtain and maintain their housing. An important implication of this study is that funding priorities should include allocating resources to programs that can be used flexibly to address survivors’ complex, multi-faceted and changing needs.

This study also has numerous implications for further research and is hopefully merely one step in continued investigations into this promising model. There are many more questions to answer about how the DVHF model works, for whom, for how long, and under what conditions. The larger longitudinal evaluation of which this study is a part can answer some of these questions, as we are collecting data every six months over two years. Additional studies are needed, however, with diverse populations, across a variety of settings, and employing various research designs. Future qualitative studies will be able to shed critical light on survivors’ perspectives, thoughts, and feelings about their situations and decisions. Studies may also be designed to examine the provision of flexible funding separately from housing advocacy. A plethora of studies are needed to determine how to effectively improve the safety and housing stability of a wide range of DV survivors.

In conclusion, this study provides promising evidence that the DVHF model leads to greater housing stability and less economic abuse for IPV survivors six months after they sought services, compared to those receiving SAU. While these initial findings are promising, it will be important to examine whether they persist over time. Further, additional group differences may emerge at different time points across the 24-months of data collection. As data from additional time points become available, we will examine different change trajectories and determine temporal causality. It is critically important to understand which interventions work best for which survivors to ensure that service providers are effectively working toward long-term housing stability and well-being for IPV survivors and their children.

## Figures and Tables

**Table 1:** Socio-Demographics of Sample at Baseline N = 406

	*n*	%
Age (*Mean* 34.5; *SD* = 9.02)		
Female	393	97
Heterosexual	350	86
Race/Ethnicity (could choose all that apply)		
Non-Hispanic White only	144	35
Hispanic/Latinx	142	35
Black	76	19
US Indigenous	48	12
Asian	16	4
Middle Eastern	5	1
Multiracial/multiethnic	62	15
U.S. Citizen	331	82
Primary Language English	324	80
Parenting Minor Children	299	74
Employed in the last 6 months	235	58
Education		
Less than high school	117	29
High school graduate / GED	49	22
Vocational /training certificate	33	8
Some college	86	21
Associate degree	28	7
Bachelor’s degree	35	9
Advanced degree	18	4
Prior history of homelessness	298	73
Homeless as a child/adolescent	87	24

**Table 2:** Significant Baseline Differences Between Those Who Received DVHF and Those Who Received Services as Usual (N = 351)

Variable	beta	Odds Ratio	SE	P	95% CI	
					Lower	Upper
1. Minority (n/y)	0.480	1.616	0.372	0.037	1.029	2.538
2. Living with abuser (n/y)	−0.892	0.410	0.179	0.041	0.174	0.964
3. Children (n/y)	0.532	1.703	0.424	0.033	1.045	2.775
4. Foster care (n/y)	−0.693	0.500	0.143	0.016	0.285	0.877
5. Inability to make ends meet	−0.164	0.849	0.061	0.023	0.737	0.977
6. Housing barriers	−0.562	0.570	0.148	0.031	0.343	0.948
7. Stayed with friends or family to avoid homelessness (as an adult) (n/y)	−0.887	0.412	0.161	0.024	0.191	0.888
8. Housing instability	−0.328	0.721	0.054	0.000	0.623	0.834
9. Seeking help with housing (n/y)	−2.128	0.119	0.124	0.041	0.015	0.916
10. Overall IPV	−0.219	0.804	0.079	0.026	0.663	0.974
11. Stalking	−0.171	0.843	0.058	0.014	0.736	0.966
12. Alcohol misuse	−0.250	0.779	0.091	0.032	0.620	0.978
13. Drug misuse	−0.247	0.781	0.074	0.009	0.649	0.940
14. Quality of life	0.237	1.268	0.123	0.015	1.048	1.535
15. Rural/urban agency	−0.938	0.391	0.091	0.000	0.248	0.618

For dichotomous variables, ”no” = 0 and ”yes” = 1. Positive beta coefficients indicate higher likelihood of receiving DVHF, while negative beta coefficients indicate higher likelihood of receiving SAU

Comparisons included those retained at 6-months (N = 345) as well as six people lost at 6-months but interviewed at 12-months

**Table 3:** IPWRA Results Comparing DVHF and SAU at Six-Months (N = 345)

Outcome	*b*	*SE*	*p-value*	CI Lower bound Upper bound		Cohen’s d
Housing stability*	−1.244	0.258	0.000	−1.751	−0.738	0.565
Economic abuse*	−0.163	0.081	0.044	−0.321	−0.004	0.194
IPV (physical, emotional, sexual abuse, stalking)	−0.112	0.064	0.081	−0.239	0.014	0.092
–Physical abuse subscale	−0.011	0.050	0.820	−0.108	0.086	−0.051
–Emotional abuse subscale	−0.105	0.081	0.195	−0.264	0.054	0.052
–Sexual abuse subscale	−0.041	0.070	0.557	−0.178	0.096	−0.015
–Stalking subscale	−0.285	0.164	0.082	−0.607	0.037	0.194

* significant at <0.05

**Table 4:** Group and Total Means and (SD) on Outcomes Baseline to Six Months

	DVHF (n = 221)		SAU (n = 124)		Total Sample (N = 345)	
	Baseline Mean (*SD*)	6-month Mean (*SD*)	Baseline Mean (*SD*)	6-month Mean (*SD*)	Baseline Mean (*SD*)	6-month Mean (*SD*)
Housing stability*	4.46 (1.67)	2.88 (1.98)	5.29 (1.54)	4.30 (1.81)	4.76 (1.67)	3.39 (2.03)
Economic abuse*	1.40 (1.06)	0.38 (0.71)	1.57 (1.04)	0.66 (1.01)	1.46 (1.05)	0.48 (0.84)
IPV (physical, emotional, sexual, stalking)	1.58 (1.09)	0.47 (0.70)	1.88 (1.19)	0.67 (0.77)	1.69 (1.11)	0.55 (0.72)
–-Physical abuse	1.22 (1.03)	0.26 (0.60)	1.41 (1.19)	0.34 (0.62)	1.29 (1.09)	0.29 (0.61)
–-Emotional abuse	1.98 (1.33)	0.51 (0.85)	2.28 (1.26)	0.72 (1.00)	2.08 (1.31)	0.59 (0.91)
–-Sexual abuse	1.08 (1.46)	0.16 (0.60)	1.30 (1.57)	0.24 (0.77)	1.16 (1.50)	0.19 (0.67)
–-Stalking	2.05 (1.55)	0.95 (1.22)	2.50 (1.70)	1.36 (1.47)	2.21 (1.62)	1.10 (1.33)

* Significant group differences p <0.05
